# Link prediction on bipartite networks using matrix factorization with negative sample selection

**DOI:** 10.1371/journal.pone.0289568

**Published:** 2023-08-16

**Authors:** Siqi Peng, Akihiro Yamamoto, Kimihito Ito

**Affiliations:** 1 Department of Intelligence Science and Technology, Graduate School of Informatics, Kyoto University, Kyoto, Japan; 2 International Institute for Zoonosis Control, Division of Bioinformatics, Hokkaido University, Hokkaido, Japan; Vellore Institute of Technology, INDIA

## Abstract

We propose a new method for bipartite link prediction using matrix factorization with negative sample selection. Bipartite link prediction is a problem that aims to predict the missing links or relations in a bipartite network. One of the most popular solutions to the problem is via matrix factorization (MF), which performs well but requires reliable information on both absent and present network links as training samples. This, however, is sometimes unavailable since there is no ground truth for absent links. To solve the problem, we propose a technique called negative sample selection, which selects reliable negative training samples using formal concept analysis (FCA) of a given bipartite network in advance of the preceding MF process. We conduct experiments on two hypothetical application scenarios to prove that our joint method outperforms the raw MF-based link prediction method as well as all other previously-proposed unsupervised link prediction methods.

## Introduction

Link prediction is the problem of predicting the absence or presence of unobserved links in a network [1, 2]. A *network* is a graph-like structure *G* = (*V*, *E*) where *V* is a set of nodes and *E* is a set of links. A link *e* ∈ *E* is defined to be a pair (*a*, *b*) where *a* and *b* are nodes from *V*. The network structure is used for modeling various real-world relational data such as social networks, airline networks, or interaction data between chemicals, genes, and diseases. In these real-world network models, however, we may have some parts of the data missing, corrupted, or unobserved [1, 2]. This leads to the need to predict whether an unobserved link should be a potential new one. Making such a prediction based on the observed data of the network is proven possible thanks to the conclusion that any two nodes connected with a link should be similar to each other in terms of the connectivity features [1–3]. For example, given a protein-protein interaction network, we can predict potential unrecorded new interactions based on the topological structure of the network for that if two proteins interact with the same group of proteins, they should also have a high possibility of interacting with each other [4]. Based on this idea, various different methods have been developed, and the task of predicting unobserved links is named the link prediction problem [1, 2]. Recently, the link prediction problem has become an extensively researched topic due to its high practical value. It has found various applications to different scenarios like recommending potential friends from a social network service [2], completing a knowledge graph [5], discovering possibly related research papers from a research network [6], or forecasting possible interactions between chemicals, genes, and diseases [7, 8].

**Fig 1:**
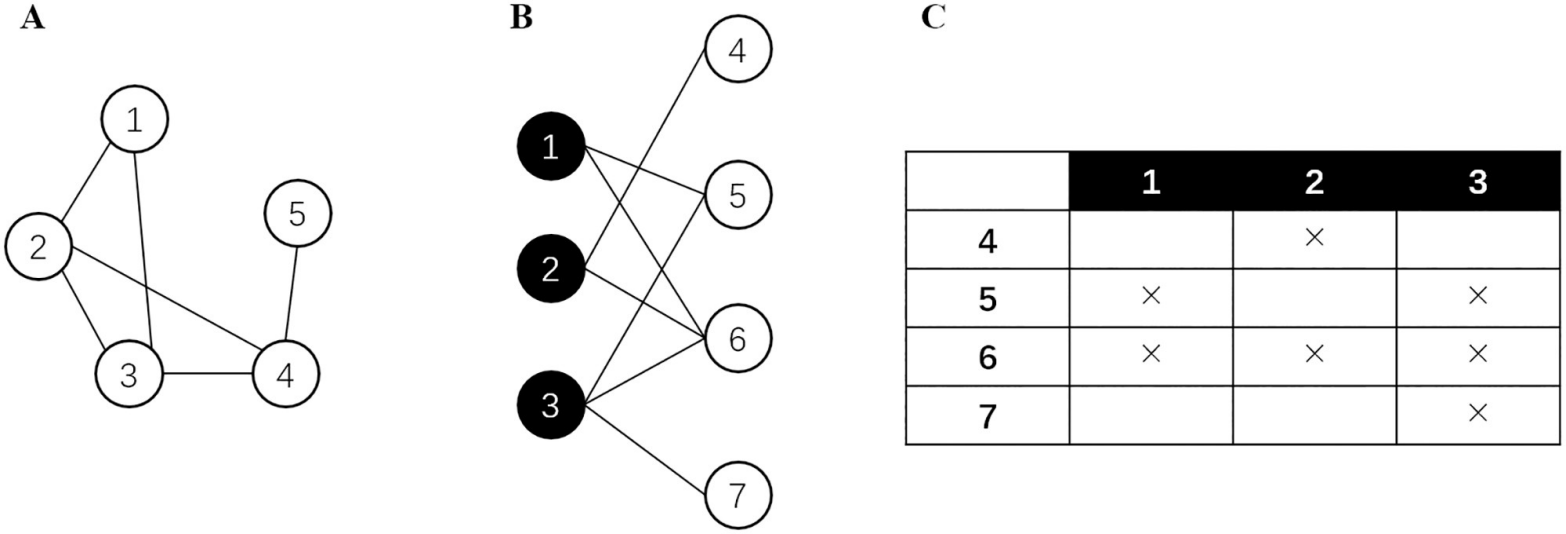
Samples of different types of networks. (A) A sample network. (B) A sample bipartite network. (C) The bi-adjacent matrix of the bipartite networks in Panel B.

A few samples of networks are depicted in Fig 1. Fig 1A shows an example of ordinary networks, which are also called unipartite networks. In a real-world network model, sometimes the nodes, *i.e.*, entities can be divided into two disjoint groups *V*_1_ and *V*_2_, where every link only connects a node from *V*_1_ to a node from *V*_2_, as is depicted in of Fig 1B [3]. This specific type of network is called a *bipartite network* [3]. The link prediction problem on bipartite networks is named *bipartite link prediction* and has been explicitly studied and discussed as an independent topic [3] for that many high-value real-world networks have the bipartite feature. It has found various applications to different scenarios such as movie recommendation [9, 10], new chemical-disease interaction detection [11] and course dropout prevention [12].

The methods for solving the link prediction problems on bipartite networks can be roughly categorized into two groups—the *unsupervised models* [13–15] and the *supervised models*. Most methods generally apply to unipartite networks, while some work better or only for bipartite networks [3]. The unsupervised methods are those applying a similarity-based scoring strategy. They are also called the *heuristic* methods [16]. They first give a similarity score for all node pairs based on the information of the observed part of the network. Then, a new link is predicted between each node pair where their similarity score exceeds a certain threshold [3, 13]. Some similarity measures are based on only the local features of the network, such as *common neighbors* (CN) [10], the *Jaccard coefficient* (JC) [13, 17], the *Adamic-Adar coefficient* (AA) [13, 18], and the *preferential attachment* (AA) [13, 19]; others are based on the global features of the whole network, such as *random walk with restart* (RWR) [20] and *PageRank* [21].

**Fig 2:**
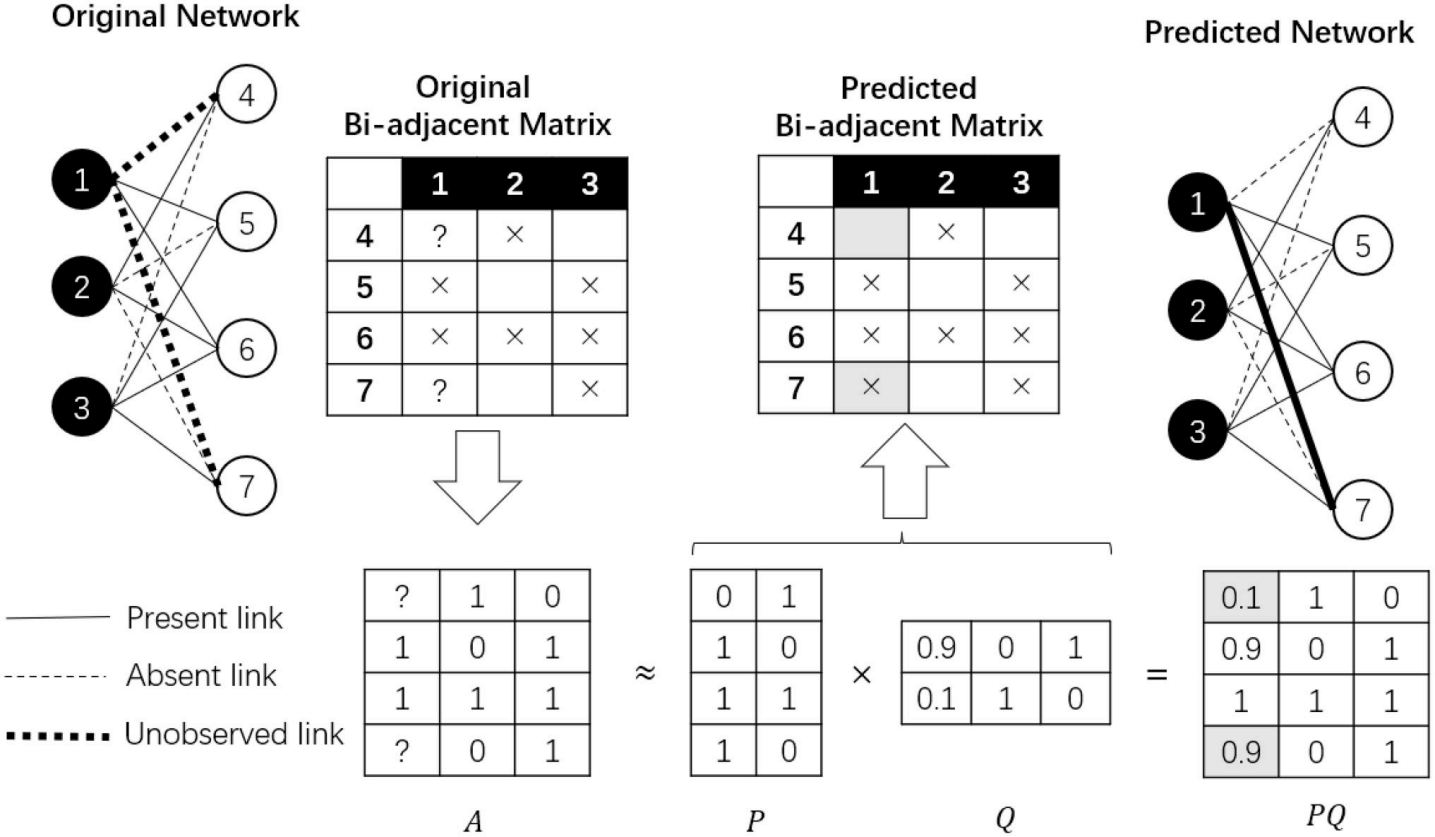
An example of the working flow of the MF-based bipartite link prediction. In the bi-adjacent matrices, present links, absent links, and unobserved links are represented with crosses, empty cells, and question marks, correspondingly. The bi-adjacent matrix of the original network is converted into a numerical matrix *A*, which is then decomposed into the product of *P* and *Q*. In the reconstructed matrix *PQ*, the confidence values of two node pairs with unobserved links (*v*_1_, *v*_4_) and (*v*_1_, *v*_7_) are 0.1 and 0.9 separately, so finally, we only predict a new link between (*v*_1_, *v*_7_).

The supervised methods, on the other hand, treat the observed part of the network as training samples and use algorithmic approaches to train a model, and use the model for predicting unobserved links [16, 22–25]. One of the most influential supervised link prediction methods for bipartite networks is *matrix factorization* (MF) [10, 11, 26, 27]. The basic idea of MF is to extract latent factors from the bi-adjacent matrix of the observed part of the network and try to reconstruct the whole network with these latent factors. Here, the bi-adjacent matrix *A* of a bipartite network *G* = (*V*_1_, *V*_2_, *E*) is a |*V*_1_| × |*V*_2_| matrix, where *a*_*ij*_, the element on the *i*-th row and *j*-th column of the matrix represents the link status between node *v*_1_ and *v*_*j*_. Given such an *N* × *M* matrix *A*, an MF-based link prediction method aims at decomposing it into the product of a *N* × *k* matrix *P* and a *k* × *M* matrix *Q*, where *k* is far smaller than *M*. Since the ranks of both *P* and *Q* are far smaller than that of *A*, the product of *P* and *Q* is considered to be a low-dimensional approximation of the original matrix, which hopefully will abstract the unnecessarily detailed information and keep the essential information of the original matrix *A*. Hence, for each node pair (*v*_*i*_, *v*_*j*_) where the link is unobserved, the corresponding value of (*PQ*)_*ij*_ is considered to be the confidence score or possibility of whether there should be a potential link. That is, a link is predicted between the node pair if the score exceeds a certain threshold. See Fig 2 for a clearer depiction of the working flow of an MF-based bipartite link prediction.

Recently with the rapid growth of computing power, supervised methods have taken up the majority of bipartite link-prediction methods [1–3, 26]. However, compared to those unsupervised methods, they rely heavily on the ground truth of the link status in the observed part of the network to create supervised data [26]. In real-world bipartite networks, there is often no ground truth of an absent link. For example, in a chemical-disease interaction network, a chemical not linked to a disease does mean that they do not interact with each other but only implies that their interaction is not yet observed. If a large number of these non-concrete absent links are treated as reliable negative training samples, it is highly likely to result in a “modest” model which tends not to predict new links at all. Such a problem has already been frequently spotted in previous research and was proved to influence prediction accuracy significantly [28, 29]. Previous solutions to the problem include utilizing side information from other data sources [6], making a minor perturbation to the data [29], and using a preliminary unsupervised scoring method for initializing or regularizing the matrix to be factorized [7, 10, 28].

In this research, we propose a new joint MF-based method utilizing a special pre-processing step called *negative sample selection* (NSS). The technique first uses a preliminary link prediction method to mark out node pairs that are **least** likely to be potential links. Then, it randomly selects a certain percentage of negative samples from the unmarked node pairs. For the preliminary link prediction method, instead of applying a traditional node-similarity-based scoring strategy, we propose a network-structure-based method derived from the *structure hole theory* [18] which gives preliminary predictions by extracting and analyzing the *overlapping bi-cliques* from the network. A similar method was proposed and studied in [13], while our method makes use of the theories and conclusions from the related field of *formal concept analysis* (FCA) to reduce the time complexity from *O*(|*C*|^2^) to *O*(|*C*|) where |*C*| represents the number of all bi-cliques and that of overlapping bi-cliques, separately. Compared to those traditional scoring methods, which give scores based on local statistic features for every single node pair, our method focuses more on extracting and comparing the overall connectivity features of node clusters, which suggests that our method is robust against the bias caused by local features and is expected to have better performance. After this well-designed negative sample selection, we can get a more reliable training set for the preceding MF process and finally get our highly accurate prediction results. We conduct experiments on three real-world datasets to simulate two hypothetical application scenarios and found that our joint method can not only work but also outperform the raw MF method as well as all other previous unsupervised bipartite link prediction methods.

## Materials and methods

### Problem formulation and evaluation

This research studies the problem of bipartite link prediction on an input network *G*_*i*_ = (*V*_*i*1_, *V*_*i*2_, *E*_*i*_) and a target network $G_{t}=(V_{t1},V_{t2},E_{t}=E_{t}^{+}\cup E_{t}^{-})$, satisfying that *V*_*i*1_ = *V*_*t*1_, *V*_*i*2_ = *V*_*t*2_ and $E_{i}\subset E_{t}^{+}$ where $E_{t}^{+}$ and $E_{t}^{-}$ represent the present links and absent links, separately. That is, the input network has the same nodes as the target network, while it only contains part of the present links and no ground truth information about absent links. The goal of link prediction is to use the information of *G*_*i*_ to build a predict network *G*_*p*_. The more *G*_*p*_ appears similar to *G*_*t*_, the more successful the system is.

To give a concise numerical evaluation, we use the following two measures: *AUC score* and *AUPR score*, which were applied in most previous research [6, 7, 11, 13, 27]. Both scores are estimated with the four basic values: TP, TN, FP, and FN. Here TP represents the number of samples that are actually positive (present) and is predicted positive; FP represents the number of samples that are negative (absent) but are falsely predicted to be positive; TN represents the number of samples that are actually negative and predicted negative; FN represents the number of samples that are actually positive but falsely predicted negative.

The AUC (Area Under the Curve) score is estimated by computing the area under the ROC (Receiver Operating Characteristic) curve. The ROC curve is created by plotting the true positive rate (TPR) against the false positive rate (FPR) at various threshold settings. Here TPR and FPR are estimated as follows:

$$\begin{array}{c} \begin{array}{cc} TPR & \overset{\text{def}}{=}\frac{TP}{TP+FN},\\ FPR & \overset{\text{def}}{=}\frac{FP}{TN+FP}. \end{array} \end{array}$$

The AUPR (Area Under the Precision-Recall curve) score is estimated by computing the area under the Precision-Recall curve, which is created by plotting the precision rate against the *recall* rate at various threshold settings. Here precision and recall are estimated as follows:

$$\begin{array}{c} \begin{array}{cc} Precision & \overset{\text{def}}{=}\frac{TP}{TP+FP},\\ Recall & \overset{\text{def}}{=}\frac{TP}{TP+FN}. \end{array} \end{array}$$

### An overview of the working flow of our method

**Fig 3:**
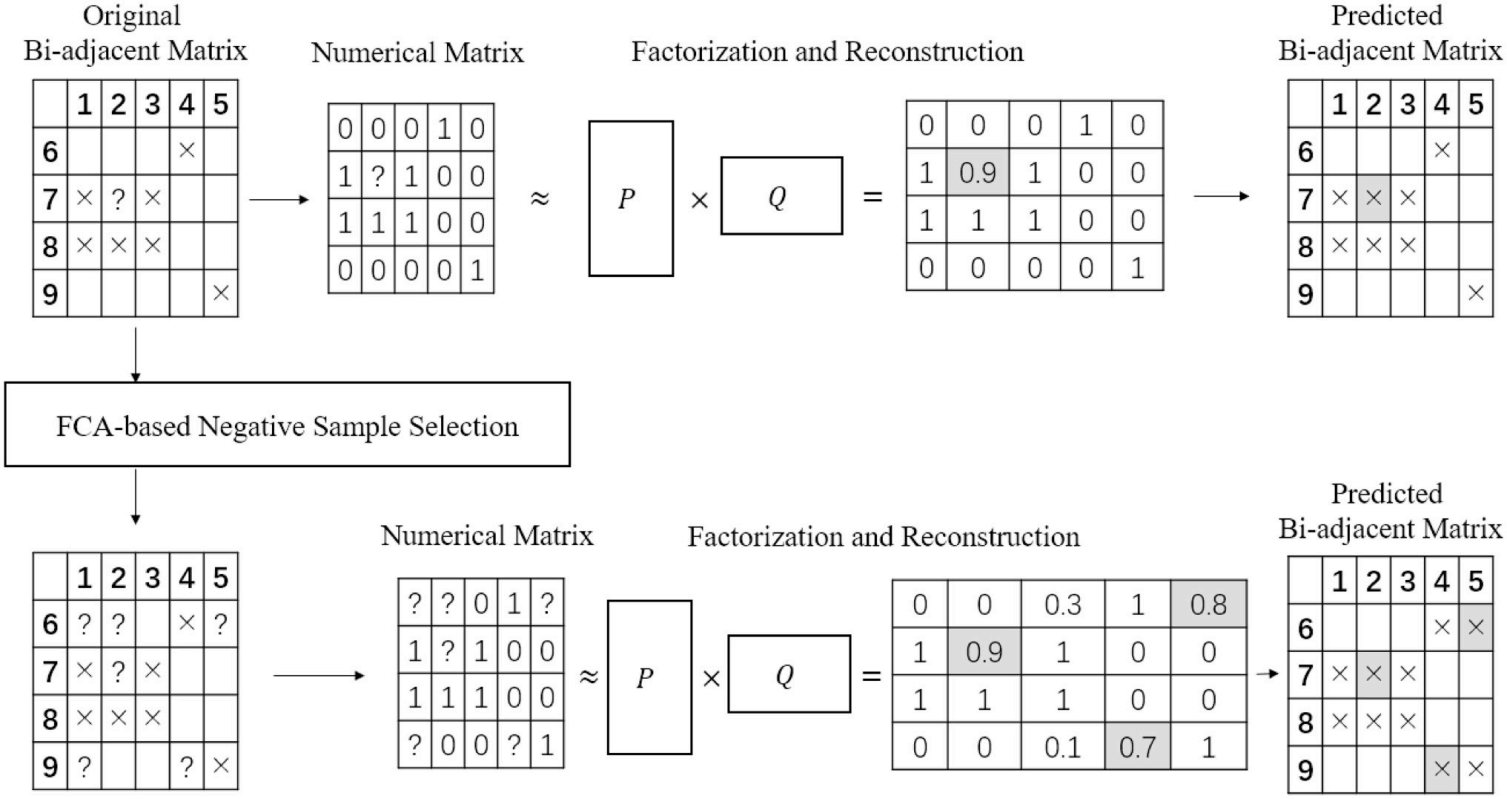
The comparison of the working flow of the raw MF method and our joint method. The upper row depicts the working flow of the raw MF method, while the lower row depicts that of our method.

Our method is named *Matrix Factorization with Negative Sample Selection* (MF-NSS). It contains two parts—the negative sample selection and the MF-based link prediction. In the first part, it processes the network and extracts maximal bi-cliques using *formal concept analysis* (FCA), a technique originally proposed for ontology extraction but also strongly connected with network theory. Then, it picks out the node pairs which are least likely to be linked together. Next, it passes the bi-adjacent matrix of the input network, with all present links marked as positive samples and the aforementioned node pairs as negative examples, to the second part. In the second part, the matrix is factorized and approximated with a collaborative filtering process, and the node pairs where their corresponding score in the reconstructed approximated matrix exceeds a threshold are outputted as our final predicted links. The comparison of the working flow of our method and the raw MF method is depicted in Fig 3.

### FCA-based negative sample selection

As introduced above, negative sample selection is the core technique of our method which selects reliable negative training samples from the input network, allowing the preceding MF process to learn more diverse latent factors. It can be thought of as a preliminary link prediction process—it roughly marks out the node pairs that are likely to be linked, and the remaining node pairs are thought to be unlikely to be linked together and thus are safe to be treated as negative training samples.

#### Overlapping maximal bi-cliques and structural hole

**Fig 4:**
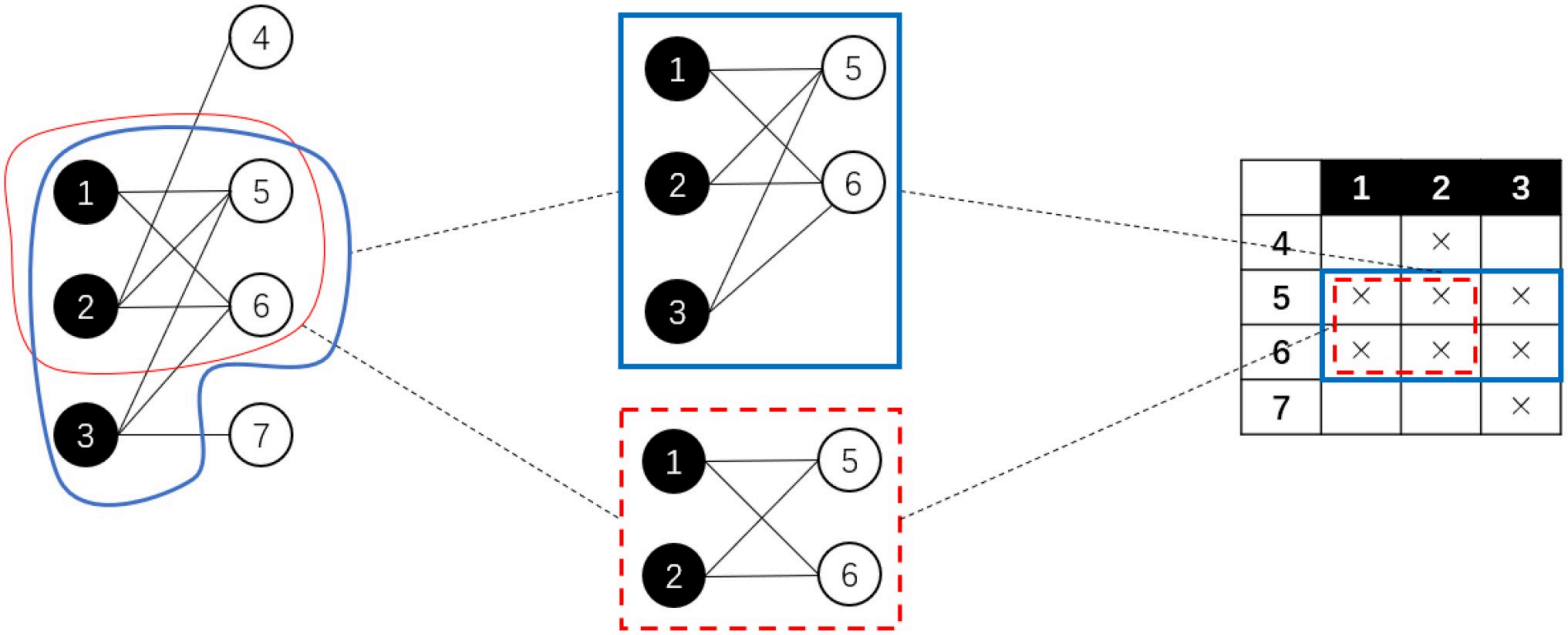
An example of a bi-clique and a maximal bi-clique. The sub-network framed out in red from the bipartite network on the left is a bi-clique, which corresponds to the rectangle framed with red dash lines in the bi-adjacent matrix shown on the right. However, it is not a maximal bi-clique because it is a sub-network of another bi-clique framed in blue. The latter bi-clique is a maximal bi-clique, for it corresponds to the rectangle framed with solid blue lines on the right, which is a maximal rectangle box filled with crosses with rows and columns permutable.

In this research, we use a preliminary link prediction method utilizing the features of *bi-cliques*. A bi-clique *C* = {*V*_*c*1_, *V*_*c*2_, *E*_*c*_} of a bipartite network *G* = (*V*_1_, *V*_2_, *E*) is a sub-network where there is a link between every node pair from different parts, that is, *V*_*c*1_ ⊆ *V*_1_, *V*_*c*2_ ⊆ *V*_2_, *E*_*c*_ ⊆ *E* and for all *v*_1_ ∈ *V*_*c*1_, *v*_2_ ∈ *V*_*c*2_ we have (*v*_1_, *v*_2_) ∈ *E*_*c*_. For such a bi-clique *C*, if no other bi-clique *D* is a super-network of *C*, then *C* is called a *maximal bi-clique*. See Fig 4 for an example of bi-cliques and maximal bi-cliques.

Bi-cliques are considered clusters of strongly-related entities. For example, in an authorship network, a bi-clique may represent a group of co-researchers and their research; in a chemical-disease network, a bi-clique represents a group of similar chemicals and their affected diseases. Intuitively, if two clusters have a lot of nodes in common, it may imply that they are both parts of a larger cluster; that is, the unlinked node pairs from two clusters should have a high possibility of being linked together. This is the famous *structural hole* theory [13, 18], which can be formalized into the following strategy for preliminary link prediction. Given a bipartite network *G*, two maximal bi-cliques *A* = {*V*_*A*1_, *V*_*A*2_, *E*_*A*_} and *B* = {*V*_*B*1_, *V*_*B*2_, *E*_*B*_} are overlapping bi-cliques if they satisfy *V*_*A*2_ ⊆ *V*_*B*2_ which mutually implies *V*_*B*1_ ⊆ *V*_*A*1_. To avoid biases, we add two extra coefficients—the component sizes of a bi-clique *A*, denoted as *s*_1_(*A*) and *s*_2_(*A*), separately; the overlapping rates of two bi-cliques *A* and *B*, denoted *σ*_1_(*A*, *B*) and *σ*_2_(*A*, *B*), separately, for measuring whether the overlapping bi-cliques are too trivial to represent a cluster of the nodes in a network:

$$\begin{array}{c} \begin{array}{cc} s_{1}\left(A\right) & \overset{\text{def}}{=}\left|V_{A1}\right|,\\ s_{2}\left(A\right) & \overset{\text{def}}{=}\left|V_{A2}\right|,\\ \sigma_{1}\left(A,B\right) & \overset{\text{def}}{=}\frac{\text{min}\{|V_{B1}|,|V_{A1}|\}}{\text{max}\{|V_{B1}|,|V_{A1}|\}},\\ \sigma_{2}\left(A,B\right) & \overset{\text{def}}{=}\frac{\text{min}\{|V_{B2}|,|V_{A2}|\}}{\text{max}\{|V_{B2}|,|V_{A2}|\}}. \end{array} \end{array}$$

**Fig 5:**
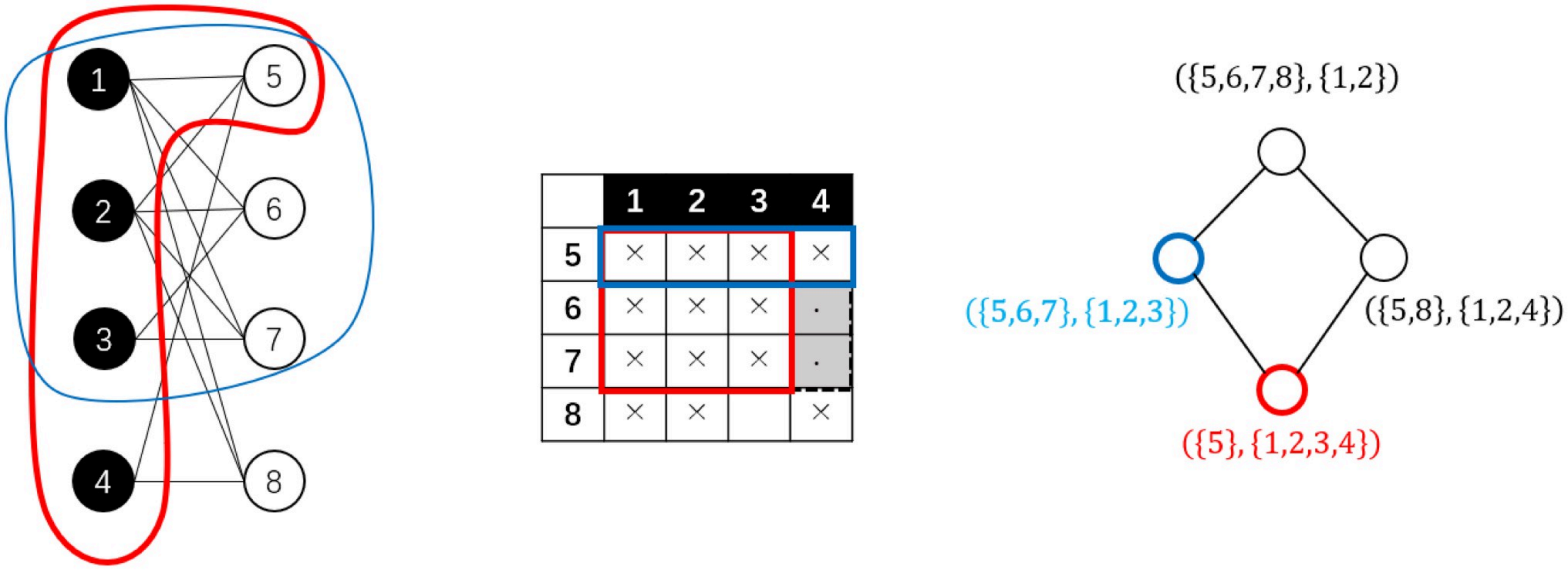
An sample pair of overlapping bi-cliques and their structure hole. Left: A bipartite network with two overlapping maximal bi-cliques framed out in red and blue, separately. Middle: The bi-adjacent matrix of the network on the left. The aforementioned maximal bi-cliques correspond to the red and blue rectangles, separately. The gray cells filled with dots represent their structure hole. Right: the concept lattice representing all maximal bi-cliques from the network. The aforementioned bi-cliques correspond to the concepts marked out in blue and red, separately.

An overlapping maximal bi-clique pair *A* and *B* is considered non-trivial if the two bi-cliques have enough sizes and overlapping rates. That is, they should satisfy the following conditions:

$$\begin{array}{c} \begin{array}{cc} \text{min}\{s_{1}\left(A\right),s_{1}\left(B\right),s_{2}\left(A\right),s_{2}\left(B\right)\} & >\alpha .\\ \text{min}\{\sigma_{1}\left(A,B\right),\sigma_{2}\left(A,B\right)\} & >\rho . \end{array} \end{array}$$

where *α* and *ρ* are threshold parameters. Based on the structure hole theory, for a non-trivial overlapping maximal bi-clique pair, all node pairs in their structure hole, denoted as *H*(*A*, *B*) are expected to have possible new links. That is, for all *v*_1_ ∈ *V*_*A*1_ − *V*_*B*1_ and *v*_2_ ∈ *V*_*B*2_ − *V*_*A*2_, a new link is predicted over each (*v*_1_, *v*_2_). See Fig 5 for a clearer view of this overlapping-based preliminary link prediction method.

Certainly, such an overlapping-based method can only weigh out some node pairs from others, which is not enough for a high-accurate link prediction method. However, it is already enough for our process of negative sample selection since we only need those node pairs that are **least** likely to be linked. That is, we simply apply this preliminary link prediction method to find out the node pairs that are **not** considered as possible links. These node pairs are marked as negative samples for the next part of our method, the MF-based link prediction, which will then extract the latent factors with these training samples and give a more accurate prediction.

#### Maximal bi-cliques and FCA

Finding maximal bi-cliques from bipartite networks is strongly connected to another task, *formal concept analysis* (FCA). FCA is a data mining technique seeking the real-world applications of mathematical order theories. It aims at extracting ontologies from a *formal context*, *i.e.*, a collection of binary relations of a group of objects and their attributes [30]. Given such a formal context, the goal of FCA is to find a maximal subset of objects and the maximal set of attributes they share in common, and these extracted ontologies are called *formal concepts*. Formally, from a formal context K=(G,M,Y), a formal concept is defined to be a tuple (*A*_1_, *A*_2_) such that

$$\begin{array}{c} \begin{array}{cc} A_{1}⊇G\; & and\;A_{2}⊇M,\\ A_{1}\times A_{2} & ⊇Y,\\ \forall X_{1}⊇A_{1}\;\forall X_{2}⊇A_{2},X_{1}\times X_{2}⊇Y & ↔X_{1}=A_{1}\;and\;X_{2}=A_{2} \end{array} \end{array}$$

where ↔ means “is equivalent to” or “stands if and only if”. Here the two components *A*_1_ and *A*_2_ in a formal concept are also called the *extent* and the *intent*, separately. This definition yields a partial order ≼ of two concepts (*A*_1_, *B*_1_) and (*A*_2_, *B*_2_):

$$\begin{array}{c} \left(A_{1},B_{1}\right)⪯\left(A_{2},B_{2}\right)\overset{\text{def}}{=}A_{1}⊇A_{2}\;and\;B_{2}⊇B_{1}. \end{array}$$

where *A*_1_ ⊆ *A*_2_ and *B*_2_ ⊆ *B*_1_ are equivalent. With such a partial order, all formal concepts from a formal context can be organized into a *concept lattice*, as is shown in Fig 5.

Clearly that if we consider the two parts of a bipartite network as the object set and the attribute set, the bi-adjacent matrix will become a formal context, and a formal concept extracted from such a formal context should represent two maximal subsets of nodes from each part of the original network where every node pairs from two different part are linked together, which completely matches the definition of a maximal bi-clique. Furthermore, a pair of overlapping maximal bi-cliques can also correspond to two concepts (*A*_1_, *B*_1_) and (*A*_2_, *B*_2_) satisfying (*A*_1_, *B*_2_) ⪯ (*A*_2_, *B*_2_), *i.e.*, a pair of concepts on the same path from the minimum concept to the maximum concept of the concept lattice, as is shown on the right of Fig 5. Furthermore, for three concepts (*A*_1_, *B*_1_), (*A*_2_, *B*_2_), and (*A*_3_, *B*_3_) satisfying (*A*_1_, *B*_1_) ⪯ (*A*_2_, *B*_2_) ⪯ (*A*_3_, *B*_3_), we have the following deductions:

- |*A*_1_| > |*A*_2_| > |*A*_3_|, |*B*_1_| < |*B*_2_| < |*B*_3_|,
- *σ*_1_((*A*_1_, *B*_1_), (*A*_3_, *B*_3_)) = *σ*_1_((*A*_1_, *B*_1_), (*A*_2_, *B*_2_)) ⋅ *σ*_1_((*A*_2_, *B*_2_), (*A*_3_, *B*_3_)),
- *σ*_2_((*A*_3_, *B*_3_), (*A*_1_, *B*_1_)) = *σ*_2_((*A*_3_, *B*_3_), (*A*_2_, *B*_2_)) ⋅ *σ*_2_((*A*_2_, *B*_2_), (*A*_1_, *B*_1_)),
- *H*((*A*_1_, *B*_1_), (*A*_3_, *B*_3_)) = *H*((*A*_1_, *B*_1_), (*A*_2_, *B*_2_)) ∪ *H*((*A*_2_, *B*_2_), (*A*_3_, *B*_3_)).

**Fig 6:**
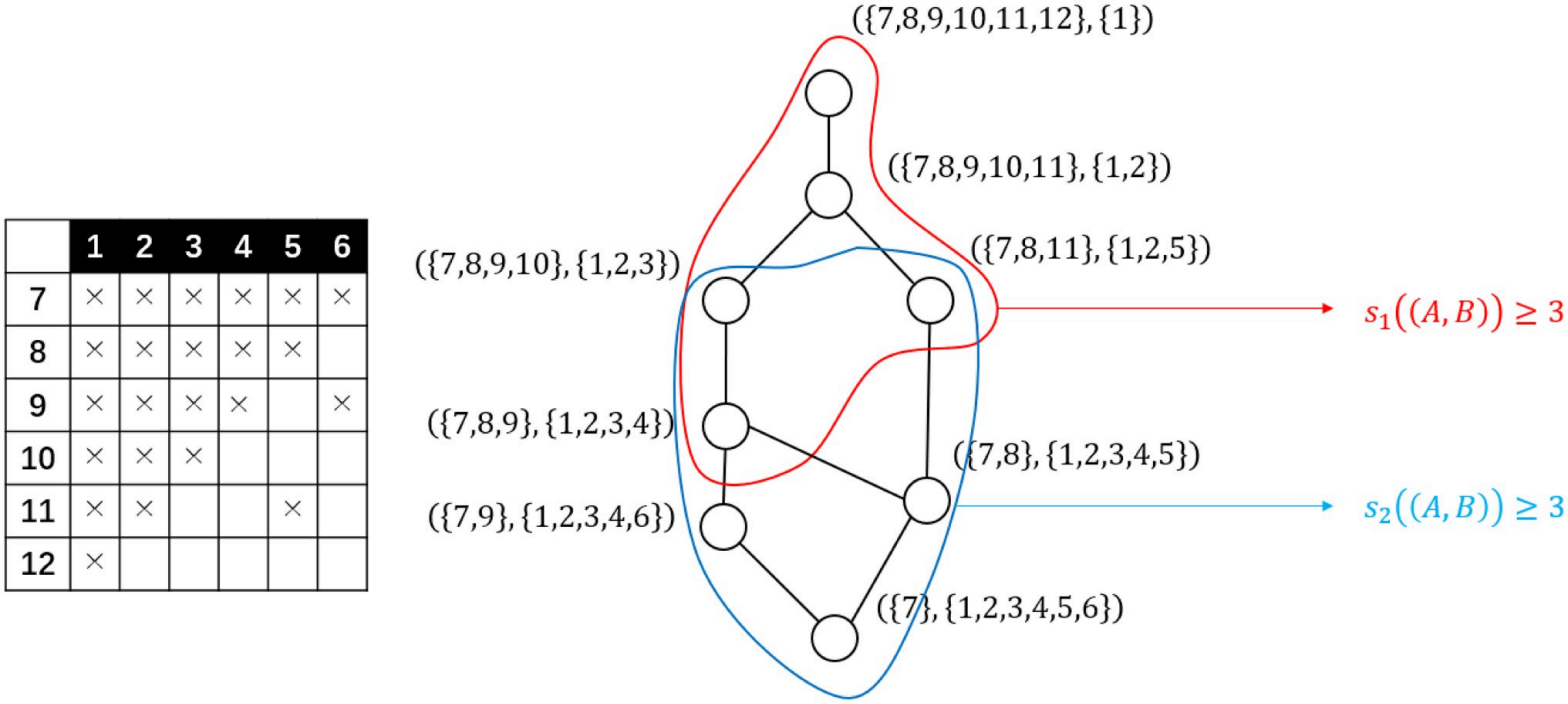
An example of the iceberg-shaped sections of the concept lattice corresponding to the bi-cliques with component sizes exceeds a threshold 3.

**Fig 7:**
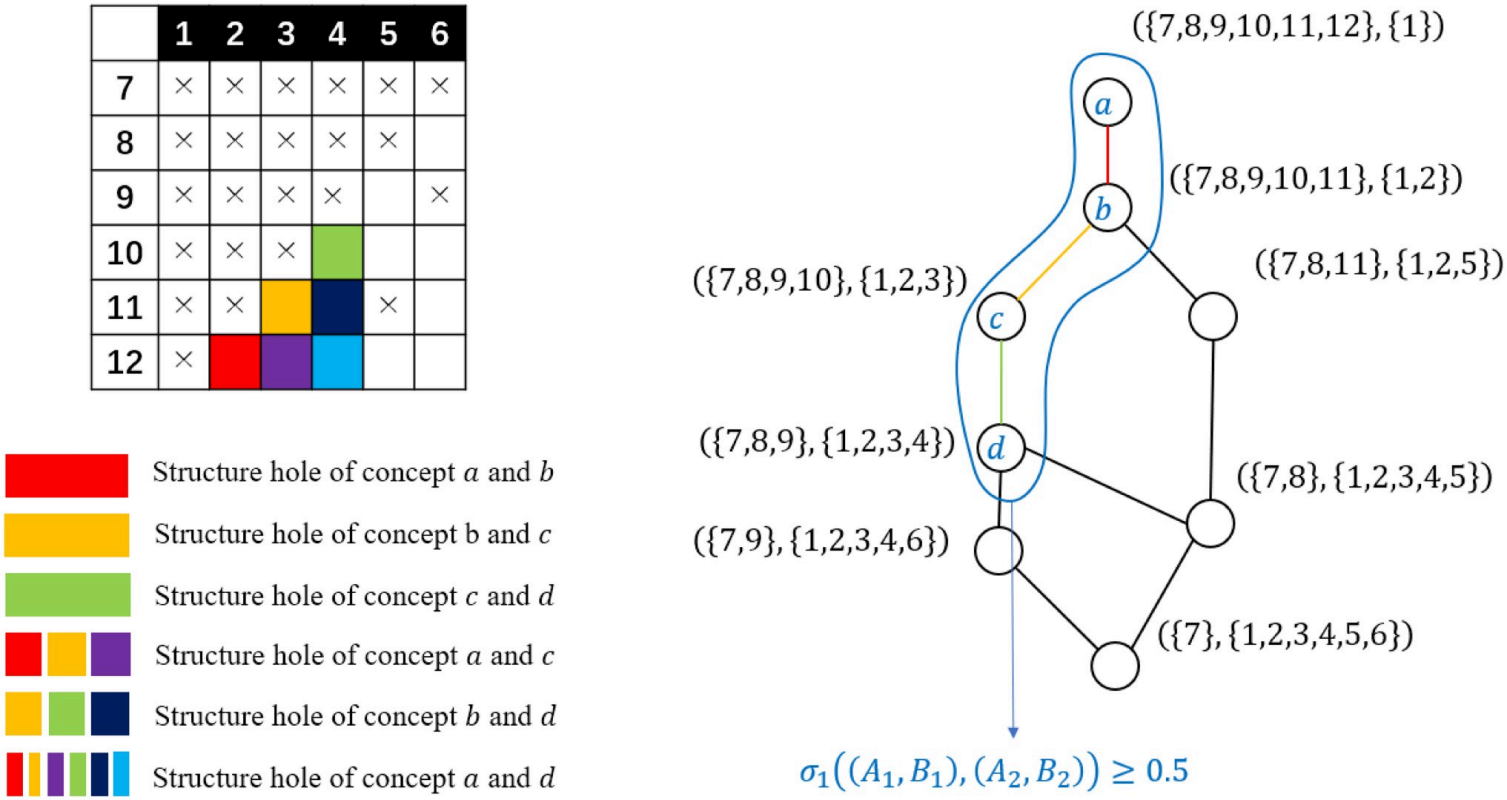
An example of a chain-shaped cluster corresponding to a group of non-trivial overlapping bi-cliques. The structure hole of each pair of concepts is marked with cells in different colors. It is clear that the structure hole of the maximum and minimum concept, *i.e.*, concept *a* and *d* contains that of any other pair of concepts from the group.

These deductions show that the component sizes and the overlapping rate of a pair of bi-cliques also correspond to the hierarchical features of the concept lattice. That is, all bi-cliques with component sizes beyond the threshold correspond to the intersection part of an upward and a downward iceberg-shaped section of the lattice. All over-lapping bi-cliques with an overlapping rate beyond the threshold correspond to several chain-shaped sections of the lattice, where the structure wholes of each pair of concepts from the section are all included in that of the minimum and maximum concept of the section. See Figs 6 and 7 for a clearer depiction of such connections. All these connections above show that to extract all non-trivial overlapping bi-cliques, all we need is to enumerate all formal concepts and construct a concept lattice from the formal context corresponding to the bi-adjacent matrix.

#### Extracting overlapping bi-cliques with FCA-related algorithms

We extract maximal bi-cliques with a modified LCM algorithm [31]. Just like most other FCA algorithms, the LCM algorithm uses an important property of formal concepts—the closure property [30]. That is, we can get a formal concept from any subset of an attribute set with the *derivation operators*. Here the derivation operators of an attribute subset *B* and an object subset *A* on a formal context $K=\{K_{1},K_{2},Y\}$, denoted as *B*^(1)^ and *A*^(2)^ correspondingly, are defined as below:

$$\begin{array}{c} \begin{array}{cc} B^{\left(1\right)} & =\{k_{1}\;|\;k_{1}\in K_{1}\;and\;\forall k_{2}\in B,\left(k_{1},k_{2}\right)\in Y\},\\ A^{\left(2\right)} & =\{k_{2}\;|\;k_{2}\in K_{2}\;and\;\forall k_{1}\in A,\left(k_{1},k_{2}\right)\in Y\}. \end{array} \end{array}$$

With these derivation operators, one can generate a formal concept (*A*^(1)^, *A*^(1)(2)^) from any attribute subset *A*. That is, given an attribute subset *A*, we first compute *A*^(1)^, all objects sharing every attribute from *A*; then we compute *A*^(1)(2)^, the maximal attribute set where every attribute is shared by all objects from *A*^(1)^; and finally, we get (*A*^(1)^, *A*^(1)(2)^), which can be easily confirmed to be a formal concept according to the definition. Although such a procedure is helpful for enumerating formal concepts, it is still not efficient enough because a formal concept can be generated from many different attribute subsets. Instead, we may consider the following *augmentation operator* *Aug*((*A*_1_, *B*_1_), *b*) which generates a concept directly from a given concept (*A*_1_, *B*_1_) and an augmentation attribute *b*:

$$\begin{array}{c} Aug\left(\left(A_{1},B_{1}\right),b\right)\overset{\text{def}}{=}\left({\left(B_{1}\cup \left\{b\right\}\right)}^{\left(1\right)},{\left(B_{1}\cup \left\{b\right\}\right)}^{\left(1)(2\right)}\right) \end{array}$$

where *b* should satisfy *b* ∉ *B*_1_. This leads to the following corollary:

**Corollary 1** For any concept (*A*_1_, *B*_1_), all its suprema (*A*_2_, *B*_2_) can be generated with *Aug*((*A*_1_, *B*_1_), *b*) by choosing a proper *b* ∈ *B*_2_ − *B*_1_.

*Proff.* Since (*A*_2_, *B*_2_) is the supremum of (*A*_1_, *B*_1_), we must have a *b* ∈ *B*_2_ − *B*_1_ such that ({*b*} ∪ *B*_1_)^(1)^ = *A*_2_ or otherwise (*A*_2_, *B*_2_) would not be a concept since $B_{2}^{\left(1\right)}⊃A_{2}$. By choosing this *b*, we should have *Aug*((*A*_1_, *B*_1_), *b*) = (*A*_2_, *B*_2_).

Now that we can generate all suprema of a concept with the augmentation operator, we can simply enumerate all formal concepts in a depth-first-search mode starting from the minimum concept (∅, ∅^(2)^). However, we may still enumerate repeated concepts simply because one concept may have multiple infima. To avoid repetitions, we need to define a topological order for all these concepts. Here we follow the idea of the LCM algorithm to define the predecessor of concept (*A*, *B*), denoted as *p*[(*A*, *B*)], as the concept (*A*_1_, *B*_1_) satisfying that min{*B* − *B*_1_} > max{*B*_1_} where max{*A*} and min{*A*} refers to the attribute included in set *A* with the maximum and minimum ID, correspondingly. In each recursive call of the depth-first-search enumeration process, only when the current concept is found to be the predecessor of the newly generated concept should we call the next recursion. More details of the algorithm can be founded in the pseudocode presented in Algorithm 1.

**Algorithm 1** The basic DFS procedure for enumerating all concepts.

1: **procedure** Enumerate ((*A*, *B*))

2:  Output (*A*, *B*)

3:  **for** *b* ∈ *M* **do**

4:   (*A*_1_, *B*_1_)←*Aug*((*A*, *B*), *b*)

5:   **if** min{*B*_1_ − *B*} > max{*B*} **then**

6:    Call Enumerate((*A*_1_, *B*_1_))

7:   **end if**

8:  **end for**

9: **end procedure**

After enumeration, we are to extract the non-trivial overlapping concepts, *a.k.a.*, non-trivial overlapping maximal bi-clique pairs with the help of the *concept lattice*. As previously analyzed, these bi-clique pairs are clustered into iceberg-shaped and chain-shaped sections in the concept lattice. Since the recursive procedure we introduce before strictly follows the hierarchical order ⪯ to traverse the lattice, recording some intermediate parameters during the recursive enumeration procedure allows us to find these clusters after the enumeration easily. Details can be found in the pseudocode presented in Algorithms 2 and 3. Note that in the pseudocodes, we use some queue-like structures, which are first-in-first-out lists supporting the following operations:

- *Q*.*push*(*A*) adds an element *A* to the back end of the list.
- *Q*.*pop*() removes the element at the front end of the list.
- *Q*.*front*() returns the element at the front end of the list.

It can be derived from the code that the recursive Enumerate function presented in Algorithm 1 will be called exactly |*C*| times, where |*C*| is the number of maximal bi-cliques in the network. After enumeration, we generate *C* non-trivial overlapping maximal bi-cliques and traverse them in Algorithm 4. Hence, the overall complexity of FCA-based negative sample selection is *O*(|*C*|), *i.e.*, linear to the number of bi-cliques from the network.

**Algorithm 2** The recursive procedure for enumerating non-trivial overlapping concepts.

 **Note** The parameter (*A*, *B*) represents the concept in process. *U* is a queue memorizing the current chain-shaped cluster of non-trivial overlapping concepts. *Sim* is a global map-like structure for recording the bounds of clusters. K=(G,M,Y) is the formal concept. *TList* is a global list of formal concepts.

1: **procedure** Enumerate ((A,B),U,Sim,K,TList)

2:  **while** *σ*_1_(*U*.*top*(), (*A*, *B*)) < *ρ* **do**

3:   *U*.*pop*()

4:  **end while**

5:  *U*.*push*((*A*, *B*))

6:  **if** |*A*| ≥ *α* and |*B*| ≥ *α* **then**

7:   *TList* ← *TList* ∪ (*A*, *B*)

8:   *Sim*((*A*, *B*)) ← *U*.*front*()

9:  **end if**

10:  **for** *b* ∈ *M* **do**

11:   (*A*_1_, *B*_1_) ← *Aug*((*A*, *B*), *b*)

12:   **if** min{*B*_1_ − *B*} > max{*B*} and |*A*| ≥ *α* **then**

13:    Call Enumerate$\left((A_{1},B_{1}),U,Sim,K,TList\right)$

14:   **end if**

15:  **end for**

16: **end procedure**

**Algorithm 3** The main procedure for enumerating non-trivial overlapping concepts.

 **Input** K=(G,M,Y), the formal context corresponding to the bi-adjacent matrix; *α*, the size threshold; *ρ*, the overlapping rate threshold.

 **Output** *List*, the list of preliminary predicted links.

1: *UList*, *DList*, *List*, *Y*_1_ ← ∅, ∅, ∅, ∅

2: Initialize *Q* as an empty queue.

3: Initialize *UpSim*, *DownSim* as empty maps.

4: Call Enumerate((∅^(1)^, ∅^(1)(2)^), *Q*, *UpSim*, (*G*, *M*, *Y*), *UList*)

5: **for** each (*m*, *g*) ∈ *Y* **do**

6:  *Y*_1_ ← *Y*_1_ ∪ (*m*, *g*)

7: **end for**

8: Call Enumerate((∅^(1)^, ∅ ^(1)(2)^), *Q*, *DownSim*, (*M*, *G*, *Y*_1_), *DList*)

9: **for** each (*A*, *B*) ∈ *UList* **do**

10:  **for** each (*v*_1_, *v*_2_) in the structure hole of (*A*, *B*) and *UpSim*((*A*, *B*)) **do**

11:   *List* ← *List* ∪ (*v*_1_, *v*_2_)

12:  **end for**

13:  **for** each (*v*_1_, *v*_2_) in the structure hole of (*A*, *B*) and *DownSim*((*A*, *B*)) **do**

14:   *List* ← *List* ∪ (*v*_1_, *v*_2_)

15:  **end for**

16: **end for**

### Matrix factorization

In this research, we are mainly to study the effect of our original technique of negative sample selection, so for this step, we only use the most basic form of an MF-based link prediction. That is, the adjacent matrix *A* of the network is approximated to a product of a *N* × *k* matrix *P* and a *k* × *M* matrix *Q*, where *K* is far smaller than *N* and *M*:

$$\begin{array}{c} A=PQ \end{array}$$

Since the ranks of *P* and *Q* are far smaller than that of *A*, the values of *P* and *Q* are not unique. Hence, to compute *P* and *Q*, we need to optimize the object function below:

$$\begin{array}{c} \underset{P,Q}{\text{min}}{∥A-PQ∥}_{2} \end{array}$$

The object function is expanded into the following form:

$$\begin{array}{c} \underset{P,Q}{\text{min}}\sum_{\left(v_{i},v_{j}\right)\neq E_{x}}{\left(a_{ij}-{\vec{p_{i}}}^{T}\vec{q_{\cdot j}}\right)}^{2} \end{array}$$

where $\vec{p_{i}}$ and $\vec{q_{\cdot j}}$ represents the row vector of the *i*-th row of matrix *P* and the column vector of the *j*-th column of matrix *Q*, correspondingly. It is clear from this form that *a*_*ij*_ is the status of a link (*v*_*i*_, *v*_*j*_) in the network, and ${\vec{p_{i}}}^{T}\vec{q_{\cdot j}}$ is our confidence score of whether it should be a new link. While this object function is enough to derive and compute the factor matrices, to improve the accuracy, the confidence score is usually added with a *bias* term:

$$\begin{array}{c} b_{ij}=\mu +b_{i}+b_{j} \end{array}$$

where *μ* is the overall average confidence score of all links, with *b*_*i*_ and *b*_*j*_ being the bias for node *v*_*i*_ and *v*_*j*_. The bias term is used to force the model to retrieve interactive features between all nodes, instead of the local features of a single node. Besides the bias, the object function is also usually added with a regularization term to prevent over-fitting:

$$\begin{array}{c} \lambda_{ij}=\lambda (b_{i}^{2}+b_{j}^{2}+∥\vec{p_{i}}{∥}_{2}+∥\vec{q_{\cdot j}}{∥}_{2}) \end{array}$$

where λ is a hyper-parameter. With these two terms added, the final object function is derived to be:

$$\begin{array}{c} \underset{P,Q}{\text{min}}\sum_{\left(v_{i},v_{j}\right)\neq E_{x}}{\left(a_{ij}-{\vec{p_{i}}}^{T}\vec{q_{\cdot j}}-b_{ij}\right)}^{2}+\lambda_{ij} \end{array}$$

The function is not convex. However, if we regard either *P* or *Q* as constant, it will become a quadratic function and can be optimized using stochastic gradient descending. Hence, to solve this optimization problem, we use a collaborative filtering strategy [9]. That is, the parameter matrix *P* and *Q* are updated alternatively in an iterative mode. In each iteration for a training sample *a*_*ij*_, we are to conduct the following four updates:

$$\begin{array}{c} b_{i}\leftarrow b_{i}+\gamma \left(e_{ij}-\lambda b_{j}\right)\\ b_{i}\leftarrow b_{j}+\gamma \left(e_{ij}-\lambda b_{i}\right)\\ \vec{p_{i}}\leftarrow \vec{p_{i}}+\gamma \left(e_{ij}\vec{q_{\cdot j}}-\lambda \vec{p_{i}}\right)\\ \vec{q_{\cdot j}}\leftarrow \vec{q_{\cdot j}}+\gamma \left(e_{ij}\vec{p_{i}}-\lambda \vec{q_{\cdot j}}\right) \end{array}$$

where $e_{ij}=a_{ij}-{\vec{p_{i}}}^{T}\vec{q_{\cdot j}}-b_{ij}$ is the predicting score for the sample, and *γ* is the learning rate, a hyper-parameter controlling the step size of each gradient descent. We repeat the iterations until every training sample is updated at least *i* times, where *i* is the pre-determined parameter for the maximum number of iterations.

After the training process is finished, we compute the confidence score for each node pair (*v*_*i*_, *v*_*j*_) ∈ *E*_*x*_ with the aforementioned equation $e_{ij}=a_{ij}-{\vec{p_{i}}}^{T}\vec{q_{\cdot j}}-b_{ij}$. We predict a new link between this node pair if the score exceeds a threshold.

From above, it can be derived that the MF process requires *i* rounds of updates and each round has about *NM* update operations. Hence, the time complexity of the MF process is *O*(*iNM*), and the overall time complexity of our MF-NSS algorithm is *O*(|*C*|) + *O*(*iNM*). For comparison, we also list the time complexity of several previous link prediction methods in Table 1. Here in the table, *D*_*e*_ refers to the average degree of a node, |*E*| refers to the number of edges. From the table, we can see that, unlike the previous methods, the time complexity of our method is strongly associated with |*C*|, the number of maximal bi-cliques with enough sizes in the network. Since |*C*| may grow exponentially as *N* and *M* increase, generally, our method is considered slower than the other methods in the list. Nevertheless, in practical cases, we can still make our method finish execution in a reasonable time since we can prevent the value of |*C*| from overgrowing by tuning the parameter *α*.

**Table 1:** The comparison of the time complexity of different bipartite link prediction methods.

Algorithm	Global or Local	Supervised or Unsupervised	Time Complexity
AA	Local	Unsupervised	*O*(*NMD*_*e*_)
JC	Local	Unsupervised	*O*(*NMD*_*e*_)
PA	Local	Unsupervised	*O*(*NMD*_*e*_)
CN	Local	Unsupervised	*O*(*NMD*_*e*_)
RWR	Global	Unsupervised	*O*(*i*|*E*|)
MF	Global	Supervised	*O*(*iNM*)
SRNMF	Global	Supervised	*O*(*NMD*_*e*_) + *O*(*iNM*)
MF-NSS	Global	Supervised	*O*(|*C*|) + *O*(*iNM*)

## Results and discussions

In this section, we examine our algorithm’s effectiveness and performance using two different scenarios of applications. We compare our algorithm with two supervised link prediction methods—raw MF and SRNMF as well as five unsupervised link prediction methods—AA, JC, CN, PA and RWR. All algorithms are written in Python and executed on a Ubuntu 18.04 System with a 2.4GHz CPU and 93.00GB RAM. The codes are available at https://github.com/MF-NSS/MF-NSS.

### Link prediction on networks without ground truth for absent links

In this experiment, we study the basic application scenario of link prediction on a network where there is no ground truth for absent links, *a.k.a*, negative samples. Such a scenario is frequently encountered in practical cases, as introduced at this paper’s beginning. However, since it is impossible to estimate the performance of the algorithms without ground truth negative samples, in this experiment, we still need to build the input and target networks from datasets with ground truth absent links available and then hide the information from the algorithms to simulate such a scenario. Hence, we choose the MovieLens 25M dataset [32] and HetRec 2011 user-rating dataset [33] to build the networks for this experiment. Both datasets have records of users’ ratings of movies from different websites, including IMDb, Rotten Tomatoes, and MovieLens. We process and build bipartite networks from these datasets with the following steps:

- First, we remove the users who rated fewer than *t* movies and the movies which are rated by fewer than *t* users from the datasets. Here *t* is set to 1000 for the MovieLens dataset and 50 for the HetRec dataset. The remaining users and movies are converted to the two components of the bipartite network, *i.e.*, *V*_*t*1_ and *V*_*t*2_.
- Then, we are to build the target network $G_{t}=(V_{t1},V_{t2},E_{t}=E_{t}^{+}\cup E_{t}^{-})$. For a user *u* and a movie *v*, if the user rates 4.5 or higher for the movie, we add (*u*, *v*) to $E_{t}^{+}$; if the user rates lower than 4.5 for the movie, we add (*u*, *v*) to $E_{t}^{-}$.
- Next, we randomly remove 20% of links from $E_{t}^{+}$ to create *E*_*i*_, *i.e.*, the link set for the input network. Especially note that in order to simulate the scenario without ground truth for negative samples, here all links in the input network are present links.

After the preprocessing, we feed the input network *G*_*i*_ = (*V*_*t*1_, *V*_*t*2_, *E*_*i*_) to the algorithms and test if they can predict the target network *G*_*t*_. For the MovieLens data, we have *V*_*t*1_ = 2675, *V*_*t*2_ = 3794 and $E_{t}^{+}=398,911$. For the HetRec data, we have *V*_*t*1_ = 1872, *V*_*t*2_ = 3261 and $E_{t}^{+}=128,657$. Since the input network generation has randomity, we conduct such a network generation five times, run the six algorithms, and collect the average results into Table 2. The standard deviations for all scores in the table are below ±0.001.

**Table 2:** The statistics of the experiment on the MovieLens and HetRec datasets.

	MovieLens		HetRec	
Algorithm	AUC Score	AUPR Score	AUC Score	AUPR Score
AA	0.619	0.142	0.546	0.160
JC	0.613	0.137	0.536	0.152
PA	0.551	0.107	0.492	0.124
CN	0.620	0.142	0.548	0.161
RWR	0.627	0.132	0.616	0.161
MF	0.779	0.358	0.703	0.291
SRNMF	0.620	0.233	0.621	0.277
MF-NSS	**0.822**	**0.396**	**0.740**	**0.324**

Bold-font numbers represent the highest performance in terms of the corresponding measurement of the corresponding dataset.

From the results, we can see that while the two supervised methods—MF and SRNMF perform better than the other five unsupervised methods on both datasets, our MF-NSS method can still further improve their performance. This suggests that the lack of information on absent links does influence the performance of supervised link prediction, while our technique of negative sample selection can lessen such a negative influence and make it possible to give more accurate predictions with limited information.

### Aggressive link prediction on networks where most links are unobserved

In this experiment, we simulate the scenario of making aggressive but reasonable hypotheses based on a small group of observed data. Such a scenario is frequently encountered in many fields like drug development—researchers may start their experiments on those chemicals predicted to be most likely to react with the interested diseases. Motivated by this, we chose the CTD chemical-disease interaction database as the dataset for this experiment. The dataset has records of two types of interactions—the *curated* ones and the *inferred* ones. The curated interactions refer to those with direct evidence published in literature curated by the CTD organization, and the inferred ones are those without direct evidence but can be inferred from external curated sources. These inferred interactions are considered important references for other research in related fields. Hence, if our algorithm can predict these inferred interactions based on the network structural features of the curated interactions only, it suggests that our algorithm may have a great ability to dig deep into the features of these networked interactions and can be used as an alternative method of inference when external curated sources are unavailable. Based on this idea, we make an input network *G*_*i*_ where only the curated interactions are collected into $E_{i}^{+}$ and make a target network *G*_*t*_ where both curated and inferred are collected into $E_{t}^{+}$ and the remaining chemical-disease pairs are collected into $E_{t}^{-}$. After processing, we have $V_{t1}=10225,V_{t2}=3283,E_{i}^{+}=103,845$ and $E_{t}^{+}=1,965,562$. We run the six algorithms five times and collect the average AUC scores and AUPR scores into Table 3. The standard deviations of all scores in the table are around ±0.001 to ±0.002.

**Table 3:** The statistics of the experiment on CTD chemical-disease database.

Algorithm	AUC Score	AUPR Score
AA	0.373	0.046
JC	0.382	0.049
PA	0.503	0.029
CN	0.388	0.052
RWR	0.500	0.021
MF	0.688	0.076
SRNMF	0.731	0.118
MF-NSS	**0.846**	**0.276**

Bold-font numbers represent the highest performance in terms of the corresponding measurement.

As can be seen in Table 3, Our MF-NSS method significantly improves both the AUC score and the AUPR score compared to all other methods. Although both the raw MF method and the SRNMF method have improved the AUC score compared to all four unsupervised methods, their AUPR scores are close to the baseline. According to previous studies [34], the AUPR score gives a more focused estimation of a model’s ability to recognize positive samples, so the low AUPR scores imply that these two methods are insensitive to present links. We analyze this because the input network in this experiment is only about 10% as dense as the networks used in the last experiment and thus has many more non-concrete absent links. Without a specially designed pre-processing procedure, these two methods will treat all these non-concrete absent links as reliable negative samples and as a result, they should have a high tendency to predict an absent link between most node pairs. On the other hand, our MF-NSS method can still make accurate predictions, thanks to the technique of negative sample selection. This perfectly shows the effectiveness of our NSS procedure and that our algorithm is suitable for such an application scenario. Also, we found that in this experiment, RWR has the lowest AUPR score among all methods that we tested, even compared to the four local methods. A similar conclusion was also mentioned in [16] that unsupervised methods may not work well in protein-protein networks since they have different link formation mechanisms than the more-common social networks or user-movie rating networks. This indicated that global methods do not always outperform local methods, but that supervised methods generally perform better than unsupervised methods.

### Studies on the performance of negative sample selection

**Fig 8:**
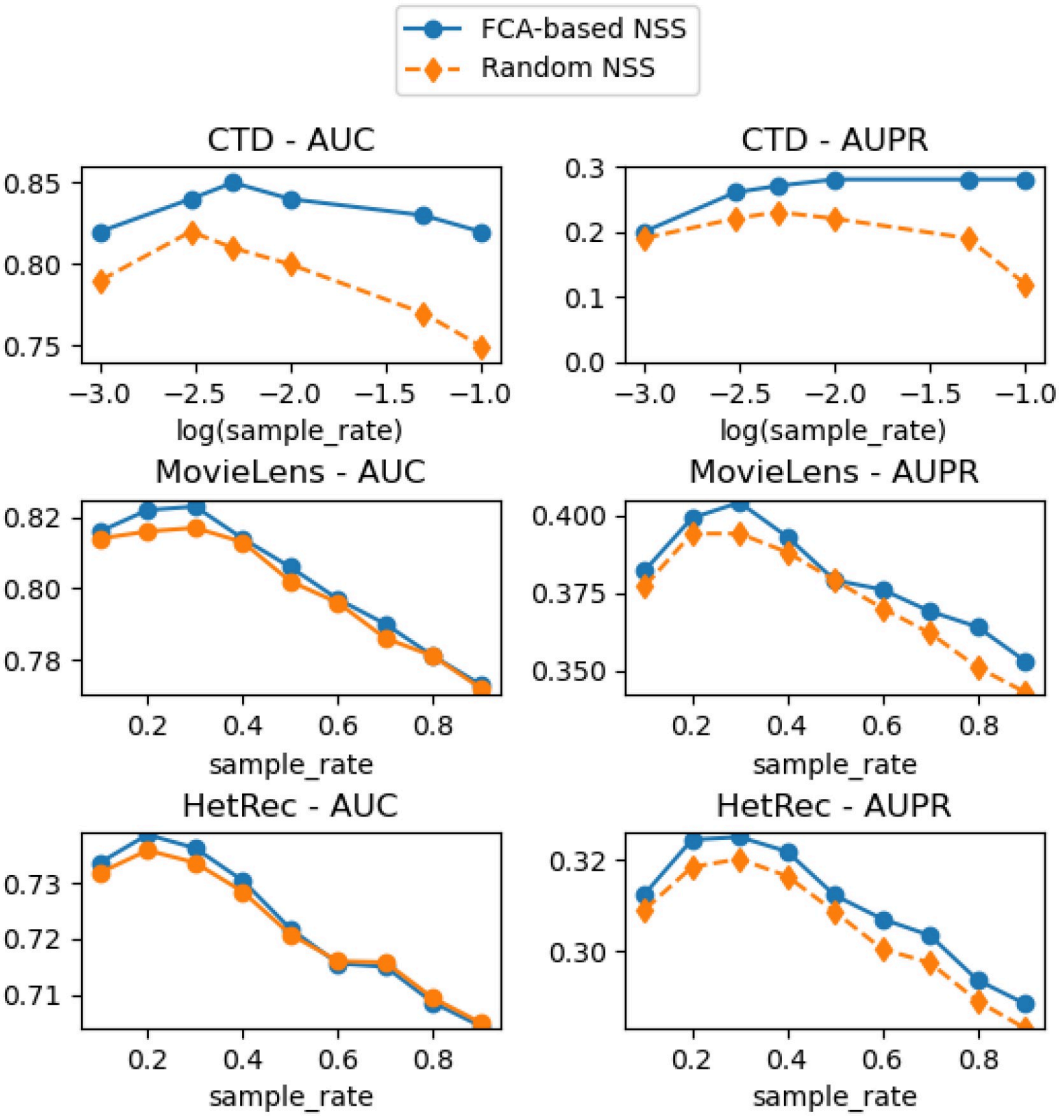
The AUC and AUPR scores with different sample rates.

In this subsection, we discuss the factors that affect the performance of the negative sample selection step. As mentioned above, in this step, we first use an FCA-based preliminary link prediction method to mark out some node pairs that are least likely to be negative samples and then randomly select a certain percentage of node pairs as negative samples from the rest of the unobserved part of the network. It can be easily derived that the parameters which control the output of the FCA-based link prediction method and the sample rate, *i.e.*, the determined percentage of the selected negative samples, should influence the final results most significantly. Hence, we design the following experiment to study how it will influence the results: First, besides the FCA-based negative sample selection procedure, we implement another procedure that completely randomly selects negative samples. Then, we run both procedures with different sample rates, feed their selected training samples into the MF procedure, and plot the final results into Fig 8.

From the results, it is clear that we may find some best sample rates contributing to the best AUC and AUPR scores—either higher or lower sample rates will result in lower scores. Also, we discover that when the sample rate is set to be these best values, both the random negative sample selection procedure and our FCA-based selection procedure can contribute to their highest AUC and AUPR scores, while the peak scores achieved by our FCA-based procedure are higher than a random selection procedure over all three datasets. This shows that our FCA-based negative sample selection procedure is effective and gives us the idea of using a random selection procedure to estimate the best sample rates before running the joint system. Furthermore, we have also found that the AUC and AUPR scores of our FCA-based negative sample selection have the largest margin over that of the random selection procedure in the task of aggressive link prediction. We analyze that it is because, in this task, there are many more positive samples in the test set, which means that a random negative sample selection will have a high probability of selecting a potential new link as a negative sample. Our FCA-based link prediction method, however, can prevent such a case from happening and thus can keep good performance in this scenario. On the other hand, in the other application scenario of link prediction without ground truth for negative samples, if there is no urgent need for high performance, it is also recommended to apply a random negative sample selection, which will slightly reduce the AUC and AUPR scores but still has a very good performance compared to a raw MF procedure or other unsupervised methods.

## Conclusion

We proposed a method called MF-NSS for link prediction. The method combines the traditional MF-based link prediction procedure with a unique FCA-based preliminary negative sample selection technique. We studied the application of this method to two typical scenarios—making aggressive predictions when most of the network is unobserved and making link predictions on a network where no ground truth for absent links is available. In the former scenario, the technique significantly improves the AUC and AUPR scores, showing the possibility of MF-NSS being used as an alternate chemical-disease or gene-disease inference method. In the latter scenario, our method can lessen the affection of excessive negative samples to reach the best performance compared to all other methods.

In our future work, we consider extending our method so that it can be further applied to heterogeneous networks, that is, networks where the links have different types. Fortunately, there has already been research on the three-dimensional extensions of formal concept analysis and matrix factorization, which will provide us with crucial theoretical basis and show us the possibility of our planned extended method. Also, we plan to develop more ways for negative sample selection and combine this technique with other supervised link prediction methods, like embedding-based methods, to see if it can boost the performance of these methods as well.
